# The effect of carbon price on low carbon innovation

**DOI:** 10.1038/s41598-023-36750-9

**Published:** 2023-06-12

**Authors:** Bernardo Cantone, David Evans, Andrew Reeson

**Affiliations:** 1 Commonwealth Scientific Industrial Research Organisation (CSIRO), 41 Boggo Road, Dutton Park, QLD 4102 Australia; 2 Commonwealth Scientific Industrial Research Organisation (CSIRO), 101 Clunies Ross St, Black Mountain Site, Canberra, ACT 2601 Australia

**Keywords:** Climate-change mitigation, Carbon capture and storage

## Abstract

Whilst many governments have implemented carbon pricing to provide firms with a greater financial incentive to develop low carbon technologies, the effect of the carbon price on the level of low carbon innovation remains unclear. In this study we develop an empirically grounded model of firms’ carbon price expectations and innovation processes. We use this model to show that a 1 USD increase in the expected future carbon price is associated with a 1.4% increase in the level of patenting in low carbon technologies, based on data for countries participating in the EU emissions trading system. We also find that firms gradually update their expectations of the future carbon price in response to recent price changes. Our findings indicate that higher carbon prices provide an effective incentive for low carbon innovation.

## Introduction

The transition towards a lower carbon economy requires the development and adoption of low emission technological innovations^1–5^. This process is already underway, with the unit costs of several low emission technologies such as photovoltaics, onshore and offshore wind, and EV batteries having fallen continuously over recent years^6^. Despite the technological advancements in these areas, further technological innovation is required to curb rising global emissions^7^. In recognition of this requirement, governments have implemented a range of policies, regulations, and R&D investments to promote the development of new low emission technologies^1–3,8^.

Carbon pricing is one of the primary mechanisms through which governments provide a financial incentive for firms to invest in developing low emission technologies^9^. Carbon pricing creates this incentive by increasing firms’ demand for low emission technologies to reduce the costs of their carbon emissions^10,11^. This increased demand for low emission technologies provides a greater potential reward for innovators who successfully develop these technologies, which in theory induces a higher level of research and development in the technologies^12^.

In recent years several countries and regions around the world have adopted carbon pricing mechanisms such as emissions trading systems (ETS) and carbon taxes^5^. One of the largest schemes in terms of coverage is the EU ETS, which has been in operation since 2005 and covers around 40% of the EU's greenhouse gas emissions^13^. The EU ETS operates in all 27 EU countries along with Iceland, Liechtenstein, and Norway (EEA-EFTA states) and covers the energy, manufacturing, and aviation sectors, with participation mandatory for most firms operating in these sectors.

Despite the theoretical prediction that higher carbon prices lead to greater investment in the development of low emission technologies^14^, there is only limited empirical evidence of the relationship between carbon prices and low carbon innovation. Econometric analysis shows that the EU ETS increased low-carbon innovation (i.e., patenting) in regulated firms versus unregulated firms by up to 10%^12^; however, low carbon prices might weaken this effect^15^. Other studies of the EU ETS find only partial evidence of the carbon price having an effect on low carbon innovation^12,16–18^; these studies focus on only a handful of firms and sectors during the two initial ETS phases (2005–2012), in which grandfathering tended to hamper low carbon investments^19^. Similarly, studies of the Chinese ETS show mixed findings, with some demonstrating that the ETS inhibited the development of low carbon technologies in the short-term^20^, whilst others show a positive impact^21,22^. Despite the widespread and increasing adoption of carbon pricing, studies are yet to establish the link between changes in the carbon price and the level of low carbon innovation over time.

This study tests the theoretical prediction that higher carbon prices cause firms to increase their levels of low carbon innovation in the short-to-medium run. To test this prediction, we use the rate of patenting in low carbon technologies as a proxy indicator of the level of innovation in these technologies. We also assume that each firm expects the future carbon price to be a moving average of recent carbon prices, as observed in studies of agents’ expectation formation processes in asset markets^23–25^. We then estimate the relationship between firms’ carbon price expectations in period $t$ and the low carbon technology patenting rate two years later, using data on all patents lodged in EU ETS member countries. Here, in line with prior research we assume a two-year lag between firms’ decisions to invest in low carbon technologies and the lodging of patent applications for these technologies (if the investment is successful)^21,22^. We use this model to infer whether higher carbon prices, through their effect on firms’ price expectations, lead to higher levels of low carbon innovation. Measuring these short-to-medium run dynamics between the carbon price, price expectations, and low emission technology development is an important step in understanding the extent to which carbon pricing can drive the technological shift required for the transition to a low carbon economy.

The rest of this article is structured as follows. The Results section presents our model of firms’ price expectations, decision-making and innovation processes which we use to link the carbon price to firms’ low carbon patenting activity. This section then applies this model to European carbon price and patenting data to estimate the relationship between the carbon price and the level of patenting in low carbon technologies. The Discussion section summarizes our main findings and their implications and provides suggestions for further related research. Finally, the Methods section details the data and modelling methods we have used in the analysis.

## Results

### A model of firms’ decision-making and innovation processes

We develop an empirically grounded model of firms’ decision-making and innovation processes to represent the mechanism through which the carbon price affects the level of patenting in low carbon technologies. This model comprises a three-step process. First, recent carbon prices influence firms’ expectations of the future carbon price. Second, these expectations of the future carbon price influence firms’ decisions about whether to invest in developing low carbon technologies. Third, if these investments are successful, firms register patents for the new technologies they have developed. Each step of this process is detailed below.

*Step 1: carbon price expectations.* In step 1 of our model, firms update their expectation of the future carbon price in response to the latest carbon price. In month $t$ firms’ expectation of the future carbon price $\tilde{p}_{t}$ is an exponentially weighted moving average of their prior price expectation $\tilde{p}_{t - 1}$ and the current price $p_{t}$:

$$\tilde{p}_{t} = \tilde{p}_{t - 1} + \alpha \left( {p_{t} - \tilde{p}_{t - 1} } \right)$$

The weight $0 \le \alpha \le 1$ determines the size of the firms’ update to their price expectations in response to $p_{t}$. Under this process, each firm has an existing price expectation $\tilde{p}_{t - 1}$, observes the new price $p_{t}$, and then updates its price expectation in the direction of $p_{t}$.

Prior research has shown that people rely on this type of anchoring and adjustment process to update their estimates of unknown quantities in a range of contexts^26^. Experimental studies have found that agents in asset markets and repeated auctions typically anchor their price expectations at a certain level and then update these expectations in the direction of the latest price change^23–25,27^, and that the above model accurately captures this process^23,27^. Further, computational studies of dynamic markets have used this model to represent agent behaviour and shown that it produces empirically accurate aggregate outcomes^28,29^.

Implementing this model requires setting a value for $\alpha$. Whilst to our knowledge there are no studies on the process through which firms update their expectations of the longer-term carbon price over time, research on investors’ price expectations in similar contexts can inform the choice of $\alpha$. Experimental studies have shown that the above model with $\alpha = 0.65$ provides a good fit for investors’ expectations of an asset’s price two years into the future when investors update their price expectations annually^23,27^. Since the firms in our model update their price expectations monthly, we set $\alpha$ as the monthly equivalent of this value. One simple way of doing this is to set $\alpha$ to make the weight assigned to the latest month’s price one-twelfth of the weight assigned to the latest year’s price in the annual updating process: $\alpha = 0.65/12 \approx 0.05$. Another approach is to set $\alpha$ to make the sum of the weights for the latest 12 months as at the current month equal to 0.65, which gives $\alpha \approx 0.08$ (see Methods section for details). Based on these calculations, we use $\alpha = 0.05$ for our baseline model and $\alpha = 0.1$ for our secondary model. Further, due to our uncertainty about how the experimental findings on price expectations translate to our study’s setting, we also test models with $\alpha \in \left\{ {0.02, 0.2, 0.5} \right\}$.

*Step 2: investment decision*. Empirical and theoretical studies of firm decision-making indicate that financial return is the central consideration in firms’ decisions about whether to invest in developing new low carbon technologies^30,31^. According to a prior model of firms’ low carbon innovation decisions, a firm invests in developing a new low carbon technology if the expected payoff exceeds the cost of the investment^30^. This expected payoff is a function of the expected carbon price and the probability of a successful investment^30^. If this probability of success and the cost of the investment remain constant, then increases in carbon price expectations lead to firms increasing their investments in new low carbon technologies.

Under our model, in each month $t$ each firm decides whether to invest in developing new low carbon technologies. Based on the above decision-making process, higher expectations of the future carbon price $\tilde{p}_{t}$ lead to firms in our model increasing their investments in developing new low carbon technologies.

*Step 3: patent application.* There is a time lag between a firm’s decision to invest in developing a new technology and the firm’s patent application for that technology (if the investment was successful). Prior research has found an average lag of 1–3 years between investment and patent application^32–35^. Based on this finding, our model assumes that if the firm’s investment in the new low carbon technology in month $t$ is successful, then the firm lodges a patent application for the new technology 2 years later (in month $t + 24$).

### Carbon prices and patenting in low carbon technologies

**Figure 1:**
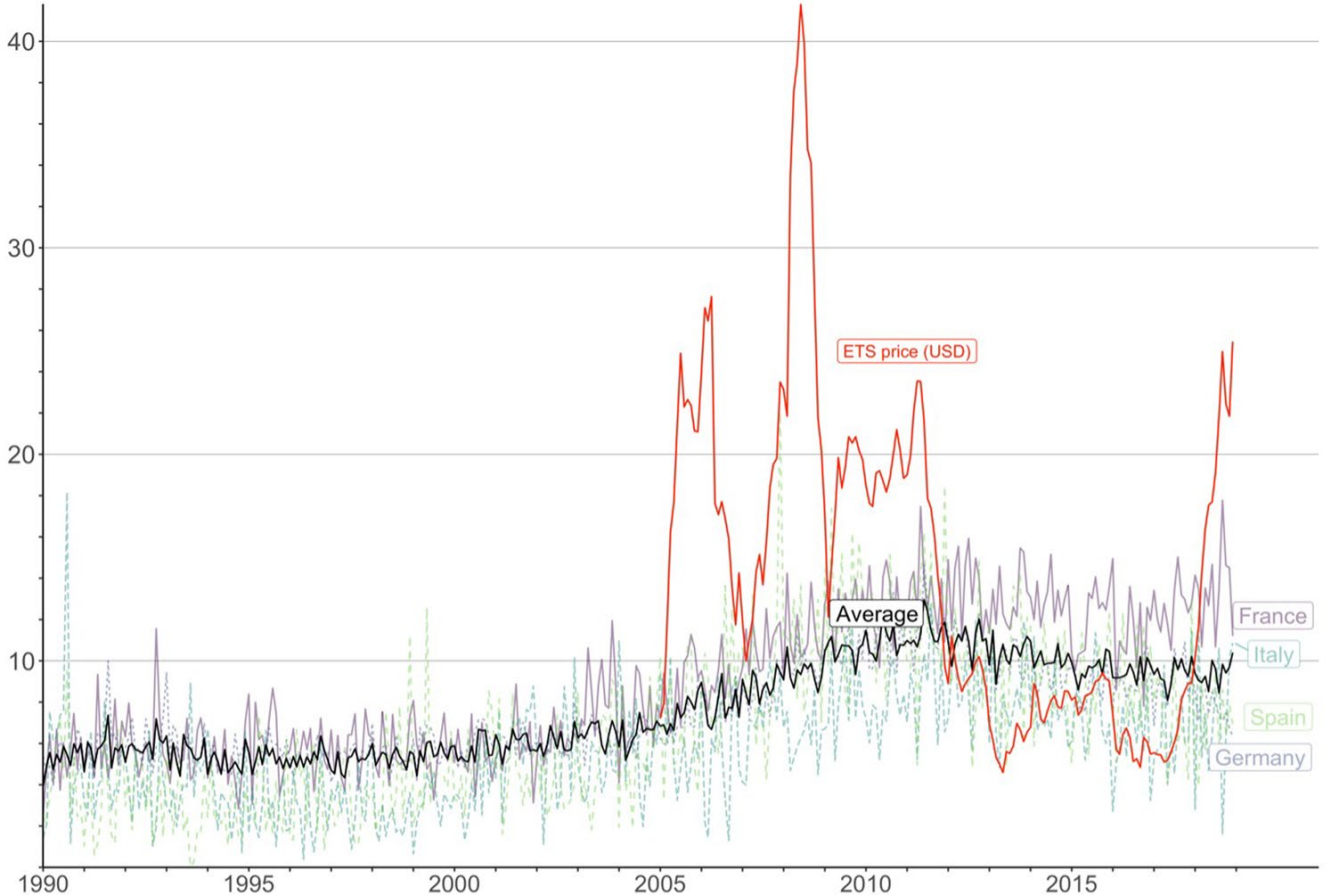
The patenting rate (%) in low carbon technologies for the 31 EU ETS countries (solid black line) and four individual EU ETS countries, along with the mean monthly EU ETS carbon price (red dashed line).

Figure 1 shows the mean monthly EU ETS carbon price from its inception in 2005 to 2019. The figure shows that the carbon price has fluctuated significantly over this period. The figure also shows the patenting rate in low carbon technologies for EU ETS countries from 1990 to 2019. We define this patenting rate as the count of patents for low carbon technologies as a proportion of all patents lodged in EU ETS countries. Here, we use the technology classes in the European patent classification (ECLA) to define low carbon technologies (see Methods section). The figure shows that in EU ETS countries low carbon technology patents accounted for about 5% of all patents in the 1990s before increasing to over 11% in 2011 and then declining to 9% in 2018.

To test the prediction that higher carbon prices cause firms to increase their levels of innovation in low carbon technologies, we analyse the relationship between the carbon price and the patenting rate in low carbon technologies between 2005 and 2018. This is the period for which we have access to EU ETS carbon price data (available from 2005 onwards) and reliable patents data (the patents database is updated over time and the data from 2019 onwards are currently incomplete).

### The effect of carbon prices on low carbon innovation

We use negative binomial regression models (1) to estimate the effect of firms’ expectation of the carbon price in month $t$ ($\tilde{p}_{t}$) on the rate of patenting in low carbon technologies in month $t + 24$ $(r_{t + 24} )$ under different assumptions about price expectations (values of $\alpha$). Here, we use negative binomial regression models (with an offset term) because the response variable is a rate, and the data indicate that the rate’s variance exceeds its expectation (i.e., there is overdispersion), such that it can be well-approximated by the negative binomial distribution. Negative binomial regression models are the standard approach to modelling this type of data^36^. Table 1 shows the regression estimates. The estimates show that in each model $\tilde{p}_{t}$ has a positive and statistically significant effect on $r_{t + 24}$, supporting the hypothesis that higher carbon prices lead to increased innovation in low carbon technologies.

**Table 1:** Estimates of the negative binomial regression of $r_{t + 24}$ on $\tilde{p}_{t}$ for under different assumptions about price expectations (values of $\alpha$).

	Model 1 ($\alpha = 0.02$)	Model 2 ($\alpha = 0.05$)	Model 3 ($\alpha = 0.1$)	Model 4 ($\alpha = 0.20$)	Model 5 ($\alpha = 0.50$)
Intercept ($\hat{\beta }_{0}$)	− 2.571***	− 2.508***	− 2.446***	− 2.403***	-2.375***
	(0.031)	(0.019)	(0.0017)	(0.017)	(0.016)
Price expectations $\tilde{p}_{t}$ ($\hat{\beta }_{1}$)	0.020*** (0.002)	0.014*** (0.001)	0.010*** (0.001)	0.007*** (0.001)	0.005*** (0.001)
Observations	144	144	144	144	144
AIC	1793.2	1764.9	1790.7	1816.5	1830.9

**Figure 2:**
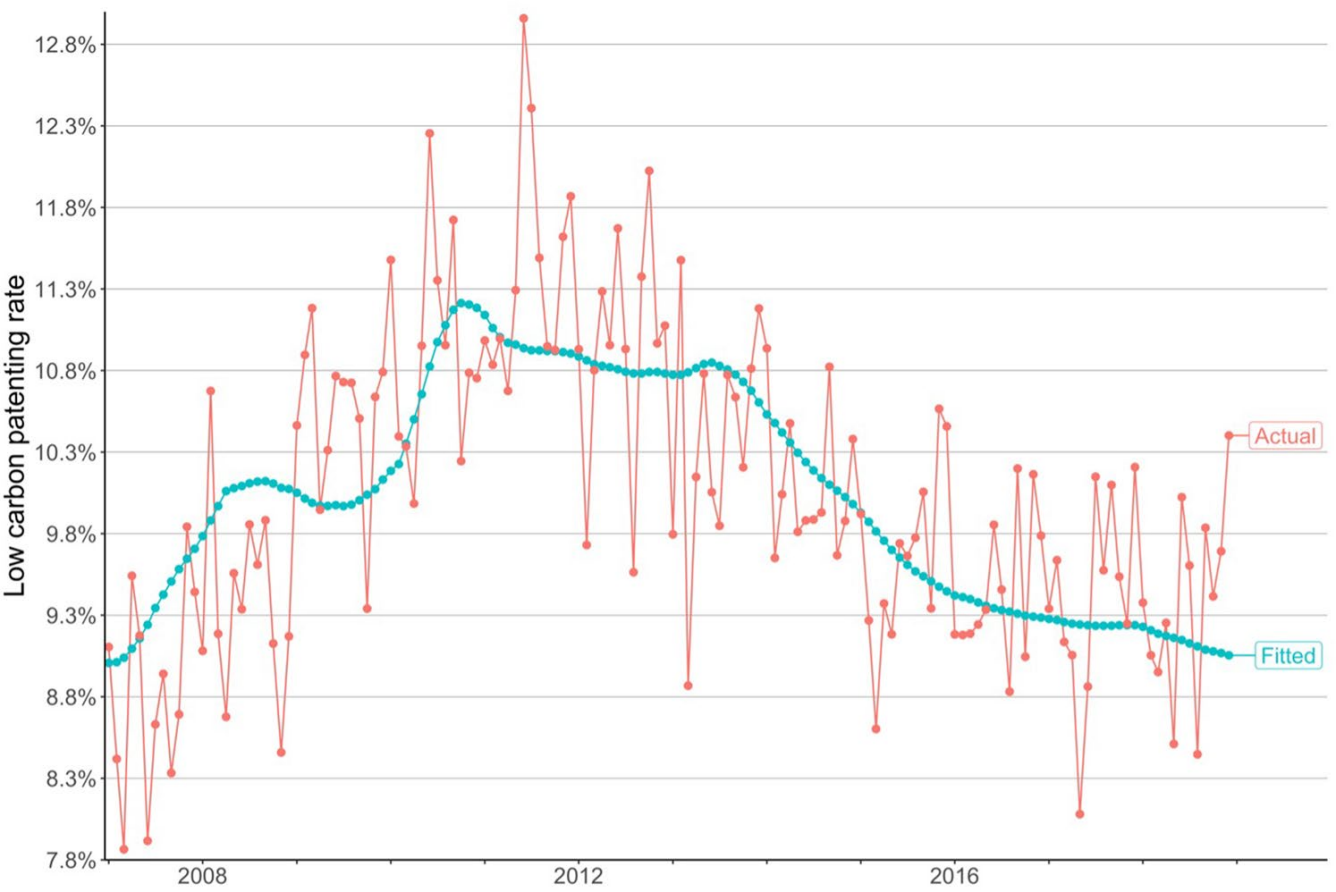
Model 2’s fitted and the actual low carbon technology patenting rates between 2007 and 2018.

The regression estimates also indicate that model 2 with $\alpha = 0.05$ has a smaller Akaike information criterion (AIC) value than the other models and therefore provides the best fit for the data. Figure 2 shows that this model explains much of the variation in the rate of low carbon technology patenting in EU ETS countries between 2007 and 2018. According to this model, a 1 USD increase in the expected carbon price in month $t$ is associated with a 1.4% increase in the rate of low carbon patents in month $t + 24$. The 95% confidence interval of this estimate is 1.1–1.7%. We have applied the Bonferroni correction to this confidence interval to account for our simultaneous testing of five hypotheses (one for each model^37^).

**Figure 3:**
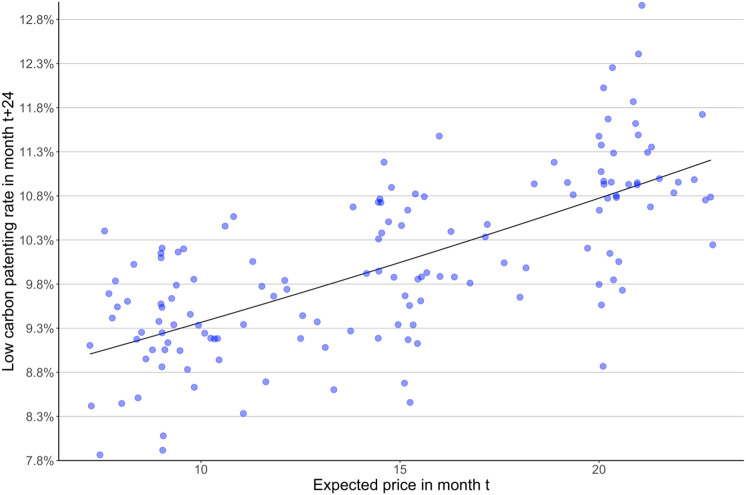
The expected and actual low carbon technology patenting rates in month $t + 24$ across different expected carbon prices in month $t$ under model 2.

Figure 3 shows model 2’s fitted (line) and the actual (points) low carbon patenting rates in month $t + 24$ across different expected carbon prices in month $t$. The figure shows that carbon price expectations vary from 7.2 USD to 22.9 USD across the years in our sample. Lower expected carbon prices of 7.2 USD are associated with low carbon technology patenting rates of 9.0% two years later, whilst higher expected carbon prices of 22.9 USD are associated with low carbon technology patenting rates of 11.2% two years later. Since the mean monthly number of patents lodged by EU ETS countries is 17,453 for the years in our sample, model 2 indicates that in an average year an expected carbon price of 7.2 USD is associated with 1,572 patents for low carbon technologies two years later, whilst an expected carbon price of 22.9 USD is associated with 1,957 low carbon patents two years later. These estimates indicate that moderate increases in carbon price expectations are associated with substantial increases in the number of patents for low carbon technologies.

As noted above, model 2 with $\alpha = 0.05$ provides the best fit for the data (lowest AIC) out of the five models. This value of $\alpha$ aligns with the values observed in experimental studies of investor behaviour in asset markets^23,27^. At $\alpha = 0.05$ firms only gradually update their expectations of the longer-term carbon price in response to the latest month’s price. That is, firms’ price expectations are sticky and depend on the carbon prices they have observed across several preceding months. Given the volatility of the monthly carbon price (see Fig. 1), this type of behaviour seems reasonable: firms have likely learned that rapidly updating their price expectations in response to the latest price fluctuation yields inaccurate expectations of the longer-term price.

### Excluding low-quality patents

In several industries firms develop large patent portfolios for very minor inventions to pursue licence fees (via litigation) from other firms who might be using or selling the claimed inventions^38,39^. Other firms have responded to these activities by developing their own portfolios of minor inventions to deter litigation from other firms through the threat of a reciprocal suit^40^. As such, changes in the rate of patenting in low carbon technologies over time might reflect this strategic behaviour by firms, rather than genuine changes in low carbon innovation.

To address this concern, we perform further analysis where we exclude low-quality patents (patents with zero citations within three years of registration) from the data set on the assumption that they potentially reflect the above strategic behaviour by firms. We then repeat the modelling and analysis on this reduced data set. This process produces similar estimates and the same conclusions as our analysis of the full data set (see supplementary information for the results).

## Discussion

We use an empirically grounded model of firm behaviour to show that higher carbon prices increase firms’ expectations of the future carbon price, leading to higher levels of patenting in low carbon technologies. Based on this model, a 1 USD increase in firms’ carbon price expectations is associated with a 1.4% increase in the number of patents for low carbon technologies two years later in EU ETS countries. This finding suggests that carbon pricing provides an effective incentive for low carbon innovation, and that significant changes in the carbon price have a substantial effect on the level of low carbon innovation.

Our analysis adds to the growing empirical research on the carbon price’s impact on innovation and in particular to those studies looking at patents as form of innovation^12,41^. Prior research found that regulated firms under the EU ETS increased their levels of patenting in low carbon technologies relative to unregulated firms, indicating that the scheme’s coverage is an important driver of low carbon innovation ^12^. We show that changes in the carbon price over time have a significant effect on the aggregate level of low carbon patenting across all firms in EU ETS countries, indicating that the price level is also a key driver of low carbon innovation.

Our model suggests that firms consider the carbon prices in the current and prior months in forming their expectations of the future price. As such, increases in the carbon price gradually affect firms’ price expectations and their decisions to invest in low carbon technologies over time. An implication of this finding is that the level of innovation in low carbon technologies is robust to short-run fluctuations in the carbon price. A further implication is that longer-run troughs or peaks in the carbon price, such as the trough in the EU ETS price between 2012 and 2017 (see Fig. 1), take time to filter through to firms’ price expectations and investment decisions.

The aggregate patents data only allow us to infer the average rate at which firms update their price expectations. We expect this rate to vary significantly across firms, with some firms rapidly updating their expectations in response to the latest carbon price and others updating their expectations far more gradually. As a result, it is likely that the trough in the EU ETS carbon price between 2012 and 2017 (see Fig. 1) caused many of the rapidly adapting firms (and/or their financiers) to revise their price expectations to very low levels and significantly reduce or cease investing in the development of low carbon technologies. Meanwhile, the gradually adapting firms likely had less disruption to their investment decisions. The average rate at which firms update their price expectations that we infer from the aggregate data masks this heterogeneity in behaviour across firms.

We identify four important areas for further research. First, we require a deeper understanding of the types of low carbon innovation that firms undertake when the carbon price rises. An analysis of patent abstracts and citations could reveal whether higher carbon prices induce greater incremental innovation in existing technologies, increased exploration of emerging technology areas, or both. This information could improve our understanding of whether higher carbon prices can induce the large technological shift required to support the transition to a low carbon economy.

Second, the analysis in this study could be expanded to explore the link between the carbon price and other forms of low carbon innovation or knowledge. Patents represent only one form of knowledge about new low carbon technologies. Academic studies represent another form of this knowledge. Estimating the relationship between the carbon price and the number of research articles about new low carbon technologies would reveal whether the carbon price has a broader effect on low carbon technology development beyond that observed in patents data.

Third, our model is limited to estimating the link between the carbon price and firms’ level of low carbon innovation at the aggregate level. Expanding our model to capture the range of firm characteristics that affect decisions to invest in developing low carbon technologies, such as the firm’s size and financial status, would provide a richer understanding of the effect of the carbon price on low carbon innovation and how this effect varies across different firms. This type of analysis would require linking patents data to other sources of data about the firms.

Fourth, research exploring how firms engaged in low carbon innovation update their carbon price expectations over time could be used to improve the model of firms’ decision-making used in this study. Based on experimental and observational studies of asset markets, we assume that firms update their expectations via an exponentially weighted moving average process. Whilst we show that this assumption explains much of the variation in the rate of low carbon technology patenting in EU ETS countries between 2007 and 2018, studying firms’ actual decision-making processes in this market could validate our assumption, reveal that firms follow a different process, or show that firms follow a variety of processes. These findings could then be used to develop a decision-making model tailored to this market.

## Methods

### Data

We obtain patents data from the European Patents Office’s (EPO) worldwide patents database PATSTAT^42^. We assign each patent in the database to a country based on the country of residence of the first applicant, which is available for 47% of the database’s patents. We then base our analysis on data for the 31 EU ETS member countries.

All patents filed at the EPO are categorized using the European patent classification (ECLA). This classification includes a technological class ‘Y02’ pertaining to “technologies or applications for mitigation or adaptation against climate change”. This class provides the most accurate tagging of climate change mitigation patents available today, represents the international standard for low-carbon innovation^43^, and has been used to analyse trends in low carbon patenting^15^. Table 2 provides codes and descriptions of the 8 technological subclasses within Y02. We define a low carbon technology patent as any patent tagged with at least one Y02 subclass^12,43^.

**Table 2:** Low-carbon technology patents classes (Y02) and subclasses.

Class	Description
Y02	Technologies or applications for mitigation or adaptation against climate change
Y02A	Technologies for adaptation to climate change
Y02B	Climate change mitigation technologies related to buildings, e.g., Housing, house appliances or related end-user applications
Y02C	Capture, storage, sequestration, or disposal of greenhouse gases [GHG]
Y02D	Climate change mitigation technologies in information and communication technologies [ICT], i.e., information and communication technologies aiming at the reduction of their own energy use
Y02E	Reduction of greenhouse gas [GHG] emissions, related to energy generation, transmission, or distribution
Y02P	Climate change mitigation technologies in the production or processing of goods
Y02T	Climate change mitigation technologies related to transportation
Y02W	Climate change mitigation technologies related to wastewater treatment or waste management

The patents database provides each patent’s citations (by other patents) along with the dates of these citations. We define any patent with zero citations within three years of registration as potentially being a low-quality patent. We then exclude these patents in our analysis in the supplementary information. The descriptive statistics of our data can be seen in Table 3.

**Table 3:** Descriptive statistics of the patents data (for 2007–2018) and carbon price data (for 2005–2018).

Variable	Obs	Mean	Std. Dev	Min	Max
Total low carbon patents ($t + 24$)	144	1,772	772	328	3,539
Total patents ($t + 24$)	144	17,453	7,082	3,883	32,911
Low carbon patents rate ($t + 24$)	144	10.00%	0.96%	7.86%	13.00%
Total high quality low carbon patents ($t + 24$)	144	280	189	1	743
Total high quality patents ($t + 24$)	144	2,437	1,543	29	5,737
High quality low carbon patents rate ($t + 24$)	144	10.80%	2.03%	1.96%	15.20%
ETS price (USD) ($t$)	168	14.50	7.97	4.59	41.80

We use ETS price data for the period 2007–2018 in our analysis. We obtain ETS allowance price (tCO2-eq) data for 2008–2018 from the International Carbon Action Partnership^44^. For the period 2005–2007 (phase 1 of the ETS) we use the price data from the European Environmental Agency^45^.

The ETS did not allow firms to carry over allowances from phase 1 (2005–2007) to phase 2 (2008–2012). As a result of this inability to carry over allowances along with an oversupply of allowances to emit in 2007, the price of an allowance to emit in 2007 decreased to low levels. At the same time, the price of an allowance to emit in 2008 onwards remained higher (both types of allowances were traded throughout 2007). Since our analysis focuses on price expectations, we use the price of future allowances (for 2008 onwards) as the carbon price throughout 2007. Note that this type of divergence between prices for allowances in different years does not arise at other points in the time series as the ETS has enabled firms to carry over allowances from one year to the next in subsequent phases.

### Regression model, statistical inference, and model selection

We use a negative binomial regression model to test the prediction that higher expected carbon prices $\tilde{p}_{t}$ lead to higher rates of patenting in low carbon technologies two years later $r_{t + 24}$. We fit the model:

$$\log \left( {E\left[ {y_{t + 24} } \right]} \right) = \beta_{0} + \beta_{1} \tilde{p}_{t} + \log \left( {z_{t + 24} } \right)$$

where $E\left[ {y_{t + 24} } \right]$ is the expected count of patents for low carbon technologies in month $t + 24$ for EU ETS countries and $z_{t + 24}$ is the total count of patents in month $t + 24$ for EU ETS countries. Here, we include the term $\log \left( {z_{t + 24} } \right)$ in the model as an offset variable to transform the (log of the) response variable into the rate of low carbon patents $r_{t + 24}$

1 $$\log \left( {\frac{{E\left[ {y_{t + 24} } \right]}}{{z_{t + 24} }}} \right) = \beta_{0} + { }\beta_{1} \tilde{p}_{t}$$

If our prediction that higher (lower) $\tilde{p}_{t}$ lead to higher (lower) $r_{t + 24} = \frac{{E\left[ {y_{t + 24} } \right]}}{{z_{t + 24} }}$ is correct, then we expect our estimates to show $\hat{\beta }_{1} > 0$.

In our analysis we fit five regression models with different assumptions about price expectations: $\alpha \in \left\{ {0.02, 0.05, 0.10, 0.20, 0.50} \right\}$. In each model we test the null hypothesis that $\beta_{1} \le 0$ (i.e., $\tilde{p}_{t}$ does not have a positive effect on $r_{t + 24}$) against the alternative hypothesis that $\beta_{1} > 0$ (i.e., $\tilde{p}_{t}$ has a positive effect on $r_{t + 24}$). Simultaneously testing five hypotheses increases the probability of making a false discovery (or type I error). To control this probability, we apply the Bonferroni correction such that we only reject each null hypothesis if $p \le \frac{\theta }{5}$, where $\theta$ is the desired overall significance level. This process ensures that the family-wise error rate (the probability of making one or more false discoveries) does not exceed $\theta$. Similarly, we adjust the confidence level for each estimate of $\beta_{1}$ to the level of $1 - \frac{\theta }{5}$ to satisfy the overall desired confidence level of $1 - \theta$.

We use the AIC to select the best fitting model of the five models. Each model’s AIC is given by:

$$AIC = 2k - 2\log \left( {\hat{L}} \right)$$

where $k$ is the number of estimated parameters in the model and $\hat{L}$ is the maximized value of the model’s likelihood function. Since all five models have $k = 2$, our procedure of selecting the model that minimizes the AIC is equivalent to selecting the model that maximizes the model’s likelihood function.

### Carbon price expectations

The expected future carbon price $\tilde{p}_{t}$ is the predictor variable in our regression model. Each firm’s $\tilde{p}_{t}$ is given by:

$$\tilde{p}_{t} = \tilde{p}_{t - 1} + \alpha \left( {p_{t} - \tilde{p}_{t - 1} } \right)$$

where $\tilde{p}_{t - 1}$ is the firm’s expectation of the future carbon price in month $t - 1$ and $p_{t}$ is the carbon price in month $t$. We initialize price expectations at $\tilde{p}_{1} = p_{1}$ in the first month of the EU ETS’s operation (January 2005) and then use the above equation to update expectations in each subsequent month.

**Figure 4:**
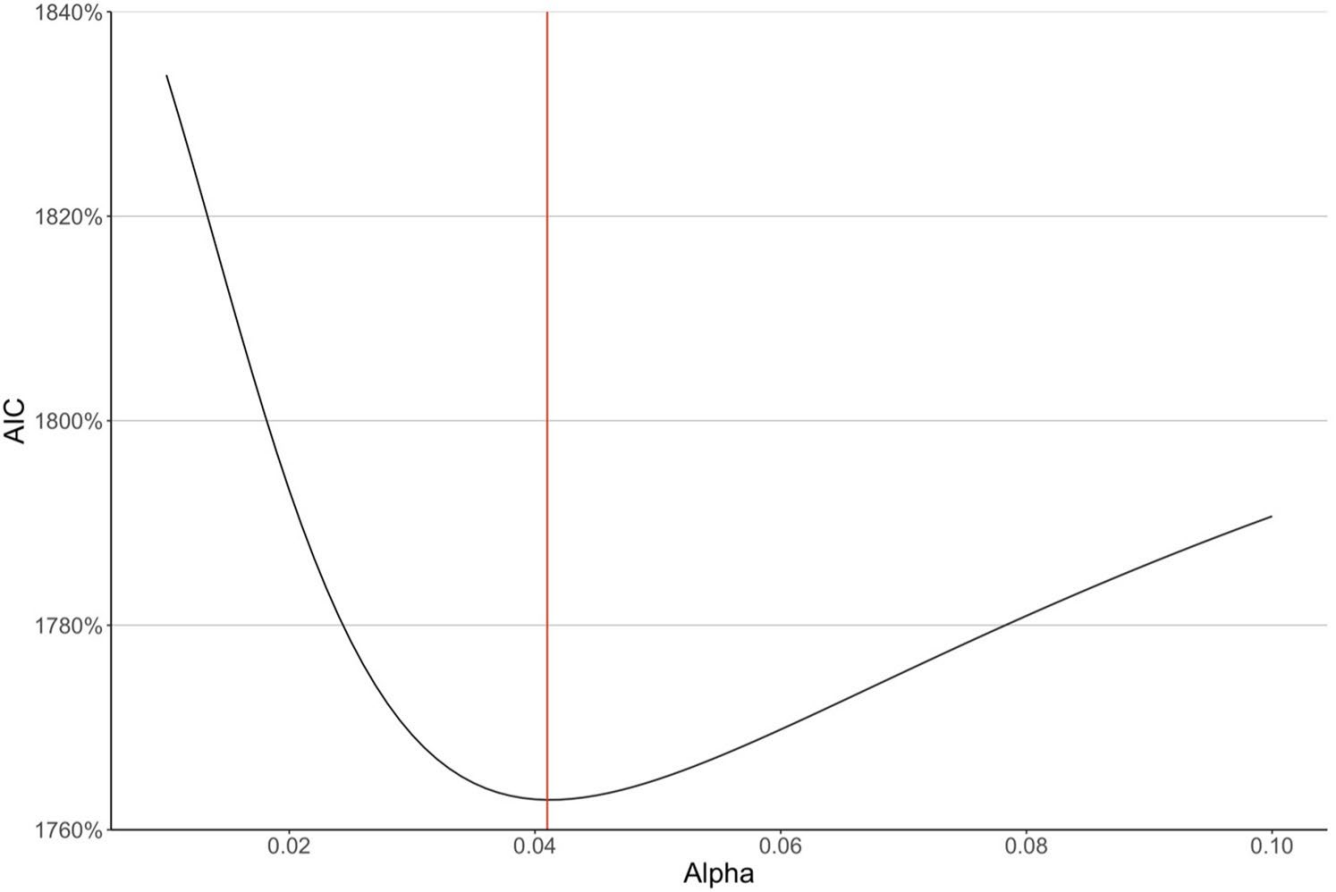
The AIC of the negative binomial regression model under $\alpha \in \left\{ {0.001, 0.002, \ldots , 0.500} \right\}$. The AIC is minimized at $\alpha = 0.041$. Deriving the monthly value of ${\varvec{\alpha}}$

The weight $0 \le \alpha \le 1$ determines the responsiveness of the firm’s price expectations to the current price. We found that the model with $\alpha = 0.05$ provided the best fit for the data (lowest AIC). We further explored the effect of $\alpha$ on the model’s fit by computing the AIC for models with $\alpha \in \left\{ {0.001, 0.002, \ldots , 0.500} \right\}$. Figure 4 shows that $\alpha = 0.041$ provides the lowest AIC, with values between 0.025 and 0.070 providing similarly low AIC values. These results indicate that firms consider both the current and prior carbon prices in forming their expectations about the future carbon price, gradually adapting their price expectations to price changes over time.

In our model of price expectations, we use the value $\alpha = 0.65$ from experimental studies where investors update their expectations annually to derive an equivalent monthly value. As noted earlier, one approach we use to do this is to set $\alpha$ to make the sum of the weights for the latest 12 months as at the current month equal to 0.65. To derive this value for $\alpha$, we first note that as at month $T$ the firm’s expectation of the future carbon price under our model can be expressed as^46^

$$\tilde{p}_{t + 1} = \mathop \sum \limits_{j = 0}^{T - 1} \alpha \left( {1 - \alpha } \right)^{j} p_{T - j} + \left( {1 - \alpha } \right)^{T} p_{0}$$

As such, the sum of the weights for the latest 12 months as at month $T$ is given by $\mathop \sum \limits_{j = 0}^{11} \alpha \left( {1 - \alpha } \right)^{j}$Then setting $\mathop \sum \limits_{j = 0}^{11} \alpha \left( {1 - \alpha } \right)^{j} = 0.65$ and solving for $\alpha$ yields our equivalent monthly value of $\alpha = 0.08$.

## Supplementary Information

Supplementary Information.

## Data Availability

The datasets generated and/or analysed during the current study are available in the GitHub repository [https://github.com/Bernaz/ETS].
