# Supplementary material for: The effect of carbon price on low carbon innovation
Source: Sci Rep. 2023 Jun 12;13:9525. doi: 10.1038/s41598-023-36750-9 (PMC10261144; doi:10.1038/s41598-023-36750-9)
Supplement: Supplementary file 1 — Supplementary Information. [file 41598_2023_36750_MOESM1_ESM.docx]

**Appendix 1: Estimates after excluding low-quality patents**

In this appendix we exclude low-quality patents (patents with zero citations within three years of registration) from the data and repeat the modelling and analysis from the main section of the report. Table A1 shows the estimates of a negative binomial regression of $r_{t+24}$ on $\tilde{p}_{t}$. The estimates show that in each model $\tilde{p}_{t}$ has a positive and statistically significant effect on $r_{t+24}$, supporting the hypothesis that higher carbon prices lead to increased innovation in low carbon technologies.

|  | | | | | |
| --- | --- | --- | --- | --- | --- |
|  | Model 1  ($\alpha=0.02$) | Model 2  ($\alpha=0.05$) | Model 3  ($\alpha=0.10$) | Model 4  ($\alpha=0.20$) | Model 5  ($\alpha=0.50$) |
|  | | | | | |
|  |  |  |  |  |  |
| Intercept ($\hat{\beta}_{0}$) | -2.340*** | -2.460*** | -2.431*** | -2.375*** | -2.332*** |
|  | (0.054) | (0.037) | (0.030) | (0.027) | (0.025) |
| Price expectations $\tilde{p}_{t}$ ($\hat{\beta}_{1}$) | 0.011**  (0.004) | 0.017***  (0.002) | 0.015***  (0.002) | 0.011***  (0.002) | 0.009***  (0.001) |
|  | | | | | |
| Observations | 144 | 144 | 144 | 144 | 144 |
| AIC | 1360.2 | 1316.3 | 1304.1 | 1318.1 | 1331.5 |
|  | | | | | |
| *Table A1: Estimates of the negative binomial regression of* $r_{t+24}$ *on* $\tilde{p}_{t}$ *under different assumptions about price expectations (values of* $\alpha$*), with low-quality patents excluded from the data. Standard errors are given in parentheses. Estimates of* $\beta_{1}$ *in models 2-5 remain statistically significant at the* $p=0.001$ *level after applying the Bonferroni correction to account for our simultaneous testing of five hypotheses (that* $\beta_{1}>0$ *in each of the five models)*^31^*. ****$p<0.001$*, ***$p<0.01$*.*  The regression estimates also indicate that model 3 with $\alpha=0.10$ has a smaller AIC than the other models and therefore provides the best fit for the data. This result differs slightly from the result for all patents in Table 1, where $\alpha=0.10$ delivered the smallest AIC. According to model 3, a 1 USD increase in the expected carbon price in month $t$ increases the rate of low carbon patents in month $t+24$ by 1.5%. The 95% confidence interval of this estimate is 1.0-1.9%. We have applied the Bonferroni correction to this confidence interval to account for our simultaneous testing of five hypotheses (one for each model^31^). | | | | | |
